# Effect of basal insulin supplement therapy on diabetic retinopathy in short‐duration type 2 diabetes: A one‐year randomized parallel‐group trial†

**DOI:** 10.1111/1753-0407.12928

**Published:** 2019-05-30

**Authors:** Pan‐Wei Mu, Xi‐Xiang Tang, Ying Tan, Yi‐Na Wang, Shuo Lin, Man‐Man Wang, Jiong Shu, Jing Wang, Yong‐Jun Zhang, Hua Liang, Bai‐Run Lin, Meng‐Yin Cai, Yan‐Ming Chen

**Affiliations:** ^1^ Department of Endocrinology The Third Affiliated Hospital of Sun Yat‐sen University Guangzhou China; ^2^ Department of Science and Technology, Guangdong Provincial Key Laboratory of Diabetology Guangzhou China; ^3^ Advanced Medical Center The Third Affiliated Hospital of Sun Yat‐sen University Guangzhou China; ^4^ Department of Endocrinology The Third Affiliated Hospital of Sun Yat‐sen University, Yuedong Hospital Meizhou China; ^5^ Department of Endocrinology The Fifth Affiliated Hospital of Zunyi Medical University Zhuhai China

**Keywords:** basal insulin, diabetic retinopathy, glycemic variability, subsequent therapy, 基础胰岛素, 糖尿病视网膜病变, 血糖波动, 后续治疗

## Abstract

**Background:**

In this study, we compared the effect on diabetic retinopathy (DR) between oral antidiabetic drugs (OADs) alone and in combination with basal insulin‐supported OADs therapy (BOT). [Correction added on 11 November 2019, after first online publication: In Abstract under Background section, “DR” has been corrected into “diabetic retinopathy (DR)”.]

**Methods:**

Between January 2015 and January 2018, this study enrolled 290 patients (age 18‐65 years) with diabetes duration between 0 and 5 years. Patients were randomly assigned to receive OADs or BOT after 14 days intensive insulin treatment. Examinations were performed at the beginning and end of the study.

**Results:**

Fewer patients developed DR in the BOT than OADs group (8 [6.06%] vs 12 [8.3%], respectively), and all cases of DR were non‐proliferative. Blood glucose concentrations were higher in the BOT than OADs group at the 3rd month, but lower in the former at the 6th and 12th month. The rate of reaching target HbA1c ≤7% was lower in the BOT than OADs group at the 3rd month (63.6% vs 72.2%, respectively), similar between the two groups at the 6th month (60.6% vs 66.6%, respectively) and higher in the BOT group at the 12th month (75.0% vs 61.1%, respectively). The SD of fasting blood glucose (FBG), coefficient of variation of FBG, SD of blood glucose (SDBG), and mean amplitude of glycemic excursions were lower in the BOT than OADs group. Changes in the levels of three cytokines (interleukin [IL]‐1β, IL‐6, and IL‐17α) were significantly less in the BOT than OADs group.

**Conclusions:**

Twelve months of BOT decreased the incidence of DR in short‐duration type 2 diabetes by reducing glycemia more effectively, stably, and completely than OADs alone.

## Graphical abstract

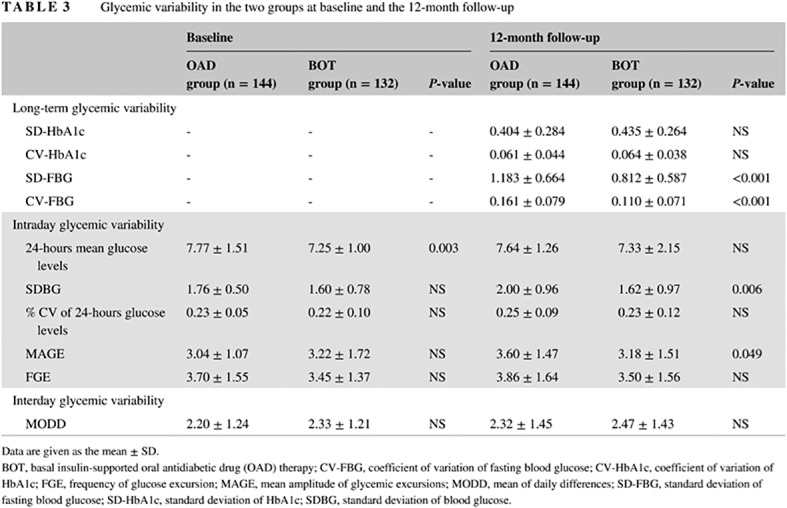

**Highlights**

- This study suggests that basal insulin‐supported oral antidiabetic drugs (OADs) therapy is superior to OADs alone in preventing diabetic retinopathy.
- This will help clinicians to select a suitable subsequent therapy after initial intensive insulin therapy.

## 摘要

**背景:**

在本研究中, 我们比较单纯口服抗糖尿病药物(oral antidiabetic drugs, OADs)和基础胰岛素联合口服抗糖尿病药物(basal insulin‐supported OADs therapy, BOT)两种治疗方案对糖尿病视网膜病变(diabetic retinopathy, DR)的影响。

**方法:**

本研究在2015年1月到2018年1月共入组290例糖尿病患者, 他们的年龄介于18到65岁之间, 糖尿病病程在0到5年之间。入组的患者先接受14天的胰岛素强化治疗, 然后被随机分入OAD组或者BOT组。在研究开始时和研究结束时进行相应的检查。

**结果:**

与OADs组相比, BOT组更少患者出现DR [BOT组8例 (6.60%) vs. OADs组12例 (8.3%)], 并且所有出现DR的患者, 其DR都属于非增殖性病变。BOT组第3个月的血糖水平较OADs组高, 但其第6个月和第12个月的血糖水平则较OADs组低。HbA1c(≤7%)的达标率, 第3个月BOT组低于OADs组(分别为63.6% 和72.2%), 第6个月两组近似(分别为60.6% 和66.6%), 第12个月BOT组高于OADs组(分别为75.0% 和61.1%)。BOT组的空腹血糖标准差、空腹血糖变异系数、血糖标准差和血糖波动平均幅度均低于OADs组。BOT组中三个细胞因子(IL‐1β、IL‐6和IL‐17α)的变化水平显著低于OADs组。

**结论:**

对于病程较短的2型糖尿病患者, 12个月的BOT方案能通过更有效、平稳、全面地降低血糖而降低DR的发生率。

## 1 INTRODUCTION

Diabetic retinopathy (DR) is a common and serious microvascular complication of type 2 diabetes (T2D) and a leading cause of blindness among the working‐age population in developed countries.^1^ As an initial treatment, intensive glycemic treatment has been shown to delay the onset and progression of DR. As subsequent treatments, both oral antidiabetic drugs (OADs) alone and basal insulin‐supported OADs therapy (BOT) are frequently administered to T2D patients. However, it remains unclear which subsequent treatment has the best effect on DR. Furthermore, it is debatable whether insulin treatment is beneficial or harmful with regard to DR. Traditionally, proliferative DR is an indication for insulin therapy, indicating that insulin is superior to OADs in treating diabetic patients with DR. However, some recent reports have claimed that insulin therapy is harmful to DR.^2^, ^3^, ^4^, ^5^, ^6^ Thus, the primary aim of this randomized controlled trial was to compare the effects of OADs and BOT as subsequent therapies on DR.

Favorable overall glycemic control is necessary to prevent the onset and progression of DR.^7^ Currently, glycemia comprises not only general glycemia, which is represented by HbA1c, but also glycemic stability, which is also called glycemic variability (GV). Because inflammation contributes to mechanisms underlying the occurrence and deterioration of DR, the secondary objective of this study was to compare the effects of these OADs alone and BOT on glycemia and inflammation.

## 2 METHODS

### 2.1 Study design and patients

This study was a single‐center prospective randomized open‐label trial conducted at the Third Affiliated Hospital of Sun Yat‐sen University between January 2015 and January 2018. The study was approved by the Ethics Committee of the Third Affiliated Hospital of Sun Yat‐sen University and the study is registered with http://clinicaltrials.gov (ID NCT02587741). Written informed consent was obtained from all participants.

The inclusion criteria were as follows: T2D diagnosis according to the 1999 World Health Organization criteria,^8^ age 18 to 65 years, body mass index (BMI) 20 to 35 kg/m^2^, duration of diabetes 0 to 5 years, and HbA1c >7.0%. Patients with a history of macrovascular disease (including ischemic heart disease, heart failure, and cerebrovascular disease), acute diabetic complications in the previous 6 months, diabetic microvascular complications, hepatic or renal impairment, malignant tumors, autoimmune diseases, and acute or chronic infections were excluded from the study, as were pregnant and lactating women.

### 2.2 Study protocol

**Figure 1:**
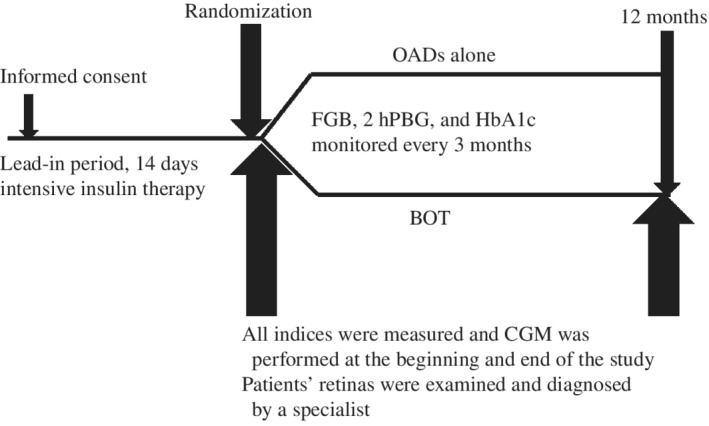
Study design. OADs, oral antidiabetic drugs; BOT, basal insulin‐supported OADs therapy; FBG, fasting blood glucose; 2hPBG, 2‐hours postprandial blood glucose

The study process is shown in Figure 1. Data for all eligible patients were collected via paper‐based case report forms and included information on baseline characteristics (sex, age, duration of diabetes, previous medical history, medications etc.) and anthropometric data (body weight, height, BMI, heart rate, and blood pressure). Urinary albumin was measured in three consecutive 24‐hour urine collections using a turbidimetric immunoassay and is expressed as the urine albumin excretion rate (UAER). For biochemical analysis, blood samples were drawn after an 8‐hour overnight fast. Laboratory assessments consisted of fasting blood glucose (FBG) concentrations, 2‐hour postprandial blood glucose (2hPBG) concentrations, liver function, renal function, lipid profiles, and electrolytes, which were measured using a HITACHI (Tokyo, Japan) 7180 Automatic Analyzer. C‐peptide and insulin were determined by radioimmunoassay (Beijing Bio‐Ekon Biotechnology, Beijing, China), whereas HbA1c was measured using the D‐10 Hemoglobin Testing System (Bio‐Rad Laboratories, Hercules, California). Serum concentrations of interleukin (IL)‐1β, IL‐6, and IL‐17α were determined using the Human Cytokine/Chemokine Magnetic Bead Panel Kit (Asbio Technology, Guangzhou, China). Eye examinations comprised visual acuity testing, tonometry, and retinal exploration. Evaluation of the retina was made by SLM‐JER slit‐lamp biomicroscopy (Chongqing Kanghua, Chongqing, China) of the posterior pole using contact lenses after pupil dilation with tropicamide 0.5% and phenylephrine HCl 10% eye drops. Retinal angiography was tested by APS‐GER digital fundus photography (Chongqing Kanghua, Chongqing, China) after intravenous injection of 10% fluorescein. Diabetic retinopathy was graded according to the International Classification of Diabetic Retinopathy.^9^ Diabetic retinopathy was diagnosed in the Department of Ophthalmology of the 3rd affiliated hospital of Sun Yat‐sen university by a single specialist, who was blinded to the treatments.

All eligible patients entered a 14‐day intensive insulin treatment lead‐in period. During the lead‐in period, continuous subcutaneous insulin infusion was performed to achieve the glycemic target, which was defined as both FBG 4.4 to 5.6 mM and 2hPBG <7.8 mM. Then, participants were randomized in a ratio of 1:  1 to either BOT or OADs as the subsequent treatment. Sealed opaque envelopes, which were arranged in a computer‐generated random order prepared by a statistician prior to the study, were opened to determine the patients' treatment assignment.

### 2.3 Treatments

#### 2.3.1 Oral antidiabetic drugs

The OADs that patients received were determined by the physicians as part of routine clinical care. The algorithm for OAD selection was one OAD from the minimum dose to the maximum dose, sequentially supplemented with additional OADs until the glucose target was achieved. The order of selection of OADs was: metformin (Glucophage; Bristol‐Myers Squibb, Shanghai, China), gliclazide (Diamicron; Servier, Tianjin, China), α‐glucosidase inhibitor (acarbose; Bayer, Shanghai, China), and a dipeptidyl peptidase (DPP)‐4 inhibitor (Januvia; Merck Sharp & Dohme, Shanghai, China).

#### 2.3.2 Basal insulin‐supported OADs therapy

Insulin glargine (Lantus; Sanofi‐Aventis, Shanghai, China) was administered initially at a dose of 0.2 IU/kg. The dose was then adjusted according to Table 1. The OADs were supplemented if the glycemic target was not achieved or maintained. The OADs were prescribed in accordance with the algorithm used for the OAD group.

**Table 1:** Algorithm for insulin dose adjustment

Fasting blood glucose (mM)	Insulin dose adjustment
<4.4	Decrease dose by 2 IU
4.4‐5.6	No adjustment required
5.6‐8.0	Increase dose by 2 IU
8.0‐10.0	Increase dose by 4 IU
>10.0	Increase dose by 6 IU

### 2.4 Outpatient follow‐up and outcome assessment

Fasting blood glucose, 2hPBG, and HbA1c values were collected every 3 months. All subjects were monitored using a continuous glucose monitoring system (MiniMed Paradigm 722; Medtronic, Northridge, California, USA) for three consecutive days at the time of randomization and at the12‐month follow‐up. At the same time, eye examinations were performed and UAER was measured. Blood samples were also collected to measure markers of inflammation. Anthropometric information, medications, self‐monitored blood glucose, hypoglycemia events, and adverse events were recorded at every follow‐up visit.

### 2.5 Glycemic control

The glycemic control in this study comprised both HbA1c to target and GV. The target HbA1c was ≤7%, and achieving this target was defined as HbA1c remission. In the present study, long‐term GV was evaluated by the standard deviation (SD) and coefficient of variation (CV) of FBG and HbA1c. The short‐term intraday GV was evaluated by the SD of blood glucose (SDBG) and the mean amplitude of glycemic excursions (MAGE). The short‐term interday GV was evaluated by the mean of daily differences (MODD), which was calculated from the mean absolute value of differences between glucose values on two consecutive days at the same time point.

### 2.6 Statistical analysis

All statistical analyses were performed using PASW statistics 19.0 (IBM Corp., Armonk, New York, USA). Continuous variables are presented as the mean ± SD, whereas categorical variables are expressed as percentages. Differences in continuous variables between groups were evaluated using Student's *t* tests, whereas differences in categorical variables were evaluated by Pearson's χ^2^ tests. Two‐sided *P* < 0.05 was considered significant.

## 3 RESULTS

### 3.1 Characteristics of study subjects

**Figure 2:**
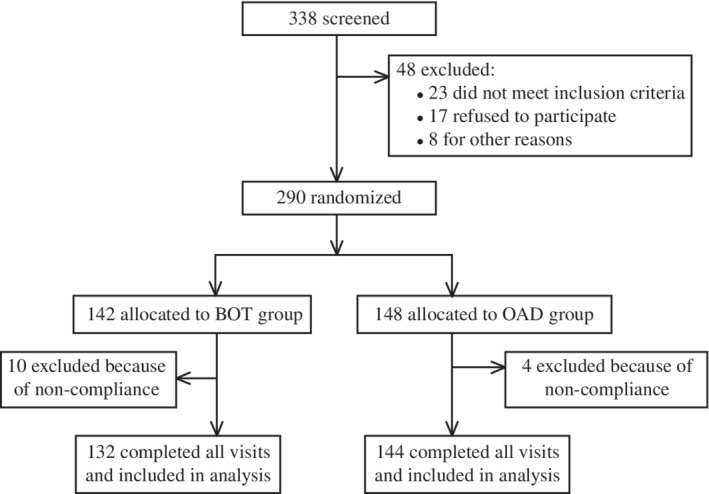
Study flow chart. OAD, oral antidiabetic drug; BOT, basal insulin‐supported OAD therapy

In all, 290 subjects were recruited in this study after the initial screening. Of these, 148 were randomly assigned to the OAD group and the remaining 142 were assigned to the BOT group. Four subjects in the OAD group and 10 in the BOT group were lost to follow‐up, all of which were in euglycemic remission during their last documented study visit (Figure 2). At baseline, no characteristics differed significantly between the OAD and BOT groups (*P >* 0.05). At the 12‐month follow‐up, UAER was significantly higher in the OAD than BOT group (*P <* 0.05); there were no significant differences in any other parameters between the two groups (*P >* 0.05; Table 2).

**Table 2:** Characteristics of all participants at baseline and at the 12‐month follow‐up

	Baseline			12‐month follow‐up		
	OAD group (n = 144)	BOT group (n = 132)	*P*‐value	OAD group (n = 144)	BOT group (n = 132)	*P*‐value
Age (y)	50.1 ± 6.7	51.7 ± 7.1	NS	‐	‐	‐
No. males/females	83/61	78/54	NS	‐	‐	‐
Diabetes duration (y)	2.3 ± 1.5	2.4 ± 2.0	NS	‐	‐	‐
SBP (mm Hg)	134.1 ± 15.4	137.0 ± 20.2	NS	130.2 ± 18.6	122.4 ± 32.3	NS
DBP (mm Hg)	85.2 ± 11.9	84.4 ± 11.7	NS	79.6 ± 8.6	80.5 ± 9.4	NS
Weight (kg)	66.08 ± 10.86	67.59 ± 10.59	NS	65.66 ± 11.00	67.30 ± 9.95	NS
BMI (kg/m^2^)	25.32 ± 2.97	25.31 ± 2.91	NS	24.47 ± 4.26	24.53 ± 2.66	NS
WC (cm)	85.97 ± 7.72	88.00 ± 9.38	NS	84.58 ± 9.82	87.81 ± 8.86	NS
FCP (pM)	1.68 ± 0.63	1.94 ± 1.88	NS	1.86 ± 0.62	1.56 ± 1.22	NS
2 h‐PCP (pM)	5.02 ± 1.97	4.19 ± 2.68	NS	5.84 ± 2.29	4.98 ± 3.71	NS
Cr (μM)	57.90 ± 20.33	58.12 ± 20.09	NS	64.16 ± 17.58	65.83 ± 20.47	NS
UA (μM)	303.58 ± 71.26	313.95 ± 84.90	NS	357.31 ± 69.42	335.62 ± 74.11	NS
TC (mM)	6.09 ± 1.58	5.59 ± 1.55	NS	5.38 ± 1.12	5.29 ± 1.21	NS
Median [25th to 75th percentile] TG (mM)	2.72 [1.31‐4.44]	2.84 [1.34‐3.77]	NS	1.88 [0.83‐2.32]	1.58 [0.86‐1.79]	NS
HDL‐C (mM)	1.48 ± 0.53	1.38 ± 0.21	NS	1.71 ± 0.71	1.64 ± 0.57	NS
LDL‐C (mM)	3.48 ± 0.77	3.27 ± 0.96	NS	2.86 ± 0.74	2.88 ± 0.93	NS
Cystatin C (μM)	0.80 ± 0.27	0.85 ± 0.39	NS	0.65 ± 0.07	0.66 ± 0.40	NS
UAER (μg/min)	5.22 ± 4.78	6.77 ± 5.34	NS	11.03 ± 8.63	5.45 ± 4.15*	0.012

Unless indicated otherwise, data are given as the mean ± SD unless.

OAD, oral anti‐diabetic drug; BOT, basal insulin‐supported OADs therapy; SBP, systolic blood pressure; DBP, diastolic blood pressure; FBG, fasting blood glucose; WC, Waist circumference; FCP, Fasting C‐peptide; 2 h‐PCP, 2 hour ‐postprandial C‐peptide; Cr, plasma creatinine; UA, uric acid; TC, total cholesterol; TG, triglycerides; HDL‐C, high density lipoprotein cholesterol; LDL‐C, low density lipoprotein cholesterol; UAER, urine albumin excretion rate.

### 3.2 Incidence of DR

At the 12‐month follow‐up, fewer patients had developed DR in the BOT than OAD group (8 [6.06%] vs 12 [8.3%]; *P* = 0.034]. The DR that developed during the follow‐up period was non‐proliferative. No diabetic macular edema was detected in any participant.

### 3.3 Glycemic control and HbA1c remission during follow‐up

**Figure 3:**
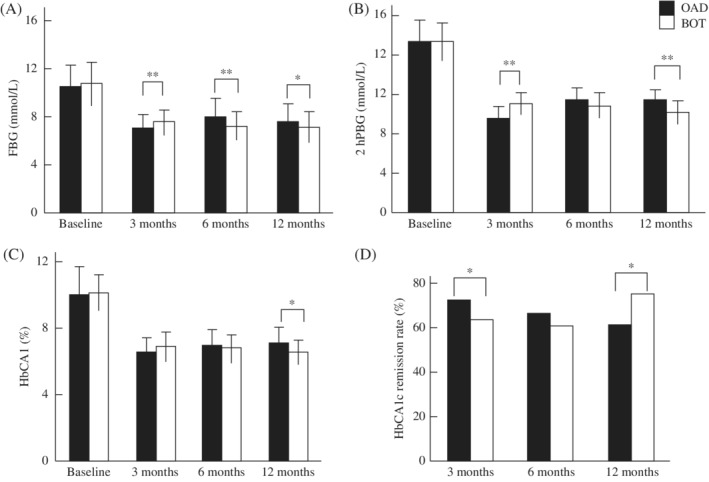
Glycemic control and proportion of patients reaching target HbA1c ≤7% in groups treated with oral antidiabetic drugs (OADs) alone or basal insulin‐supported OAD therapy (BOT). Data are the mean ± SD. *P < 0.05, **P < 0.01 (Student's *t* test). FBG, fasting blood glucose; 2hPBG, 2‐hours postprandial blood glucose

The results of FBG, 2hPBG, and HbA1c monitoring during the 12‐month follow‐up period are shown in Figure 3A–C. At baseline, there were no significant differences in FBG, 2hPBG, or HbA1c between the two groups (*P >* 0.05). At the 3rd month, FBG, 2hPBG, and HbA1c were higher in the BOT than OAD group (*P <* 0.05). However, at both the 6th and 12th month, FBG, 2hPBG, and HbA1c were all lower in the BOT than OAD group (*P <* 0.05).

After 3 months of treatment, fewer patients had achieved the HbA1c target (≤7.0%) in the BOT than OAD group (63.6% [84/132] vs 72.2% [104/144], respectively; *P <* 0.05). However, among those who achieved the HbA1c target at the 3rd month, fewer patients in the BOT than OADs group failed to maintain the target at the 6th month (n = 4 vs 8). This led to a similar HbA1c remission rate between the two groups at the 6th month (60.6% and 66.6%, respectively; *P >* 0.05). At the 12th month, the HbA1c remission rate increased to 75.0% (99/132) in the BOT group, but declined further to 61.1% (88/144) in the OAD group. This difference was statistically significant (*P <* 0.05; Figure 3D).

### 3.4 Glycemic variability between the two groups

With regard to long‐term GV, neither SD‐HbA1c nor CV‐HbA1c differed between the two groups (*P >* 0.05), whereas both SD‐FBG and CV‐FBG were significantly lower in the BOT than OAD group (*P <* 0.001). With regard to intraday GV, SDBG and MAGE were both significantly lower in the BOT than OAD group (*P <* 0.05). With regard to interday GV, neither FGE nor MODD differed significantly between the two groups (*P >* 0.05), as presented in Table 3.

**Table 3:** Glycemic variability in the two groups at baseline and the 12‐month follow‐up

	Baseline			12‐month follow‐up		
	OAD group (n = 144)	BOT group (n = 132)	*P‐*value	OAD group (n = 144)	BOT group (n = 132)	*P‐*value
Long‐term glycemic variability						
SD‐HbA1c	‐	‐	‐	0.404 ± 0.284	0.435 ± 0.264	NS
CV‐HbA1c	‐	‐	‐	0.061 ± 0.044	0.064 ± 0.038	NS
SD‐FBG	‐	‐	‐	1.183 ± 0.664	0.812 ± 0.587	<0.001
CV‐FBG	‐	‐	‐	0.161 ± 0.079	0.110 ± 0.071	<0.001
Intraday glycemic variability						
24‐hours mean glucose levels	7.77 ± 1.51	7.25 ± 1.00	0.003	7.64 ± 1.26	7.33 ± 2.15	NS
SDBG	1.76 ± 0.50	1.60 ± 0.78	NS	2.00 ± 0.96	1.62 ± 0.97	0.006
% CV of 24‐hours glucose levels	0.23 ± 0.05	0.22 ± 0.10	NS	0.25 ± 0.09	0.23 ± 0.12	NS
MAGE	3.04 ± 1.07	3.22 ± 1.72	NS	3.60 ± 1.47	3.18 ± 1.51	0.049
FGE	3.70 ± 1.55	3.45 ± 1.37	NS	3.86 ± 1.64	3.50 ± 1.56	NS
Interday glycemic variability						
MODD	2.20 ± 1.24	2.33 ± 1.21	NS	2.32 ± 1.45	2.47 ± 1.43	NS

Data are given as the mean ± SD.

BOT, basal insulin‐supported oral antidiabetic drug (OAD) therapy; CV‐FBG, coefficient of variation of fasting blood glucose; CV‐HbA1c, coefficient of variation of HbA1c; FGE, frequency of glucose excursion; MAGE, mean amplitude of glycemic excursions; MODD, mean of daily differences; SD‐FBG, standard deviation of fasting blood glucose; SD‐HbA1c, standard deviation of HbA1c; SDBG, standard deviation of blood glucose.

### 3.5 Inflammatory biomarkers

**Figure 4:**
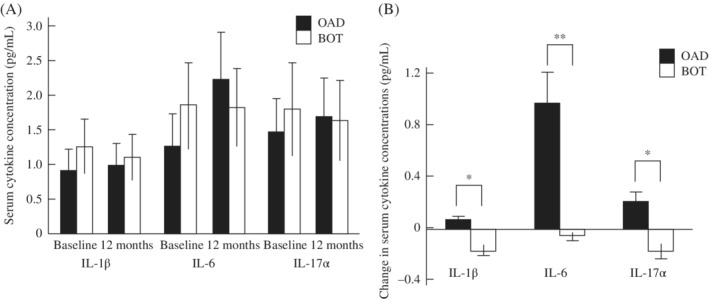
A, Serum cytokine concentrations and B, changes in serum cytokine concentrations in patients receiving oral antidiabetic drugs (OADs) alone or basal insulin‐supported OAD therapy (BOT). A, There were no significant differences in serum concentrations of interleukin (IL)‐1β, IL‐6, or IL‐17α between the two groups at baseline or after 12 months. B, However, there were significant differences in changes in serum cytokine concentrations between the two groups. Data are the mean ± SD. *P < 0.05, **P < 0.01 versus OAD group (Student's *t* test)

In the present study, IL‐1β, IL‐6, and IL‐17α were selected as inflammatory biomarkers. There were no significant differences in any of these cytokines at baseline or at the 12‐month follow‐up between the two groups (Figure 4A). However, all changes in IL‐1β, IL‐6, and IL‐17α were significantly smaller in the BOT than OAD group (Figure 4B).

### 3.6 Adverse events

There were no serious adverse events in either group. No significant changes in body weight were observed in either group (Table 2). Similar rates of hypoglycemia were observed in the OAD and BOT groups (9.87% vs 10.71%, respectively; *P >* 0.05).

## 4 DISCUSSION

The results of the present study indicate that, as a subsequent treatment, the BOT regimen was associated with a reduced occurrence of DR than OADs alone at the 12‐month follow‐up. The BOT regimen maintained the HbA1c target longer than OADs alone, with similar rates of hypoglycemia. In addition, the BOT regimen reduced long‐term and intraday GV compared with OADs alone.

Our results regarding the reduction in the incidence of DR with basal insulin‐supplemented therapy agree with those of previous studies. A few reports have indicated that insulin treatment prevents or delays DR in type 1 diabetes (T1D).^10^, ^11^, ^12^, ^13^, ^14^ A systematic review revealed that insulin treatment can also reduce DR in T2D, although the reduction was not as marked as that in T1D.^15^ The findings of an experimental animal study also suggested that insulin treatment benefited DR.^16^ However, some studies have suggested that insulin therapy could be harmful with regard to DR.^2^, ^3^, ^4^, ^5^, ^6^ These inconsistent results imply that the relationship between insulin treatment and DR is complicated. Indeed, numerous factors contribute to this relationship, such as the duration of diabetes, HbA1c, background treatment, complications, accompanying diseases, β‐cell function, the timing and duration of insulin therapy, and the stage of DR. To the best of our knowledge, no investigation has compared the effects on DR of different regimens as subsequent therapies after intensive glycemic treatment. The findings of the present study suggest that BOT is superior to OADs alone as a subsequent therapy to prevent or delay DR, which implies that basal insulin supplementation benefited DR. Given that both BOT and OADs alone are widely used in clinical practice, further targeted studies are required.

Favorable overall glycemic control is necessary to prevent the onset of DR and to delay its progression.^7^ Currently, HbA1c is considered the gold standard for general glycemic control and it has been shown to be closely related to diabetic complications, such as DR.^10^, ^11^, ^12^, ^13^, ^14^, ^15^ The results of the present study showed that HbA1c levels were lower in the BOT than OAD group at both the 6th and 12th month, although they were higher in the BOT than OAD group at the 3rd month. This result suggests that basal insulin supplementation provides a longer and better‐maintained glycemic target, resulting in a lower incidence of DR. Notably, glycemia includes not only HbA1c but also GV. Recently, GV has emerged as one of the components of glycemia.^17^ The Diabetes Control and Complications Trial (DCCT) was the first study that suggested that GV should be considered. In the DCCT study, the DR risk was substantially lower in the intensive treatment group than in the conventional treatment group (6% vs 14%), although the HbA1c values were approximately 9% in both groups.^18^ This difference was attributed to GV. Numerous later studies have further indicated that GV plays an important role in the development and progression of DM complications.^19^, ^20^, ^21^, ^22^, ^23^, ^24^ Hence, to prevent the occurrence of DR, optimized antidiabetic therapy should focus on not only HbA1c, but also GV. Glycemic variability is more complex than general glycemia. Unfortunately, there has been no gold standard to measure GV until now. Glycemic variability at a minimum includes long‐term GV, short‐term intraday GV, short‐term interday GV, and the incidence of hypoglycemia. In the present study, we used SD‐FBG, SD‐HbA1c, CV‐FBG, CV‐HbA1c, SDBG, MAGE, and MODD to represent GV. As shown in Table 3, most of the indices of GV were lower in the BOT than OAD group. We therefore suggest that BOT can decrease glycemia more completely than OADs. At the same time, the incidence of hypoglycemia in the BOT group was similar to that in the OAD group. Overall, the BOT regimen decreased glycemia more effectively, stably, and completely than OADs without increasing hypoglycemia, which may contribute to its superiority in the prevention of DR.

Inflammation is a major pathogenic factor associated with DR.^25^, ^26^ Both general hyperglycemia and GV can induce and enhance inflammation.^27^, ^28^, ^29^, ^30^ Therefore, we compared the levels of selected cytokines between the two treatment regimens. Among the cytokines, IL‐1β, IL‐6, and IL‐17α have been reported to contribute greatly to DR,^31^, ^32^, ^33^, ^34^, ^35^, ^36^ although there is some controversy regarding this issue.^37^, ^38^ Thus, we measured these three cytokines in the present study. Unfortunately, none of the cytokines showed any differences between the two groups at the 12‐month follow‐up. Notably, the changes in serum concentrations of all cytokines between the 12‐month follow‐up and baseline differed significantly between the two treatment regimens. The only difference between the BOT and OADs regimens was basal insulin supplementation. Consequently, the differences in the changes in cytokines may be due to a direct anti‐inflammatory effect of insulin, which is independent of its hypoglycemic effect.^39^

Interestingly, UAER, which is usually used to screen for diabetic nephropathy (DN), decreased in the BOT group and increased in the OAD group at the 12‐month follow‐up. These results suggest that basal insulin supplementation also benefits DN. Given that both DN and DR are characteristic microvascular complications of T2D, the results indicate that basal insulin supplementation benefits the microvascular complications of diabetes.

This study had some limitations, the most important of which is that the follow‐up time was relatively short. Diabetic retinopathy is a chronic complication of diabetes that usually appears in patients with poorly controlled glycemia of a long duration. A 12‐month follow‐up may not be sufficient, which may have contributed to the fact that few DR cases occurred in either group (8/132 in the BOT group vs 12/144 in the OAD group) and that all cases of DR were non‐proliferative. Prospective studies with longer follow‐up periods are required to validate this 12‐month result. Because there were few cases of DR in the present study, the sample size appears to be relatively small, and hence studies with a larger sample size are needed. In this study, there were no differences in cytokine concentrations between the two groups at the 12‐month follow‐up. Consequently, the significance of differences in the changes in IL‐1β, IL‐6, and IL‐17α between the two regimens was relatively slight. Therefore, we cannot determine whether these three cytokines were responsible for the superiority of basal insulin supplementation. More in‐depth studies with expanded cytokine measurements are required to further investigate the mechanisms underlying the effects of BOT.

### 4.1 Conclusion

In conclusion, the findings of this study suggest that 12‐month basal insulin supplementation as a subsequent therapy decreases the incidence of DR in short‐duration T2D by reducing glycemia more effectively, stably, and completely.

## DISCLOSURE

None of the authors has any conflicts of interest to declare.

## References

[1] Solomon SD , Chew E , Duh EJ , et al. Diabetic retinopathy: a position statement by the American Diabetes Association. Diabetes Care. 2017;40:412‐418. 28223445 10.2337/dc16-2641 PMC5402875

[2] Zhao C , Wang W , Xu D , Li H , Li M , Wang F . Insulin and risk of diabetic retinopathy in patients with type 2 diabetes mellitus: data from a meta‐analysis of seven cohort studies. Diagn Pathol. 2014;9:130 https://doi.org./10.1186/1746-1596-9-130. 24972631 10.1186/1746-1596-9-130 PMC4227060

[3] Hu L , Li DH . Relationship between modified homeostasis model assessment/correlative serum factors and diabetic retinopathy among type 2 diabetics with insulin therapy in Guangzhou, China. Int J Ophthalmol. 2014;7:463‐468. 24967192 10.3980/j.issn.2222-3959.2014.03.14 PMC4067660

[4] Kuo JZ , Guo X , Klein R , et al. Association of fasting insulin and C peptide with diabetic retinopathy in Latinos with type 2 diabetes. BMJ Open Diabetes Res Care. 2014;2:e27. 10.1136/bmjdrc-2014-000027 PMC4212555 25452868

[5] Zhang J , Ma J , Zhou N , Zhang B , An J . Insulin use and risk of diabetic macular edema in diabetes mellitus: a systemic review and meta‐analysis of observational studies. Med Sci Monit. 2015;21:929‐936. 25816765 10.12659/MSM.892056 PMC4384512

[6] Jingi AM , Noubiap JJ , Essouma M , et al. Association of insulin treatment versus oral hypoglycaemic agents with diabetic retinopathy and its severity in type 2 diabetes patients in Cameroon, Sub‐Saharan Africa. Ann Transl Med. 2016;4:395. 27867947 10.21037/atm.2016.08.42 PMC5107390

[7] Tripathy D , Chavez AO . Defects in insulin secretion and action in the pathogenesis of type 2 diabetes mellitus. Curr Diab Rep. 2010;10:184‐191. 20425581 10.1007/s11892-010-0115-5

[8] Alberti KG , Zimmet PZ . Definition. Diagnosis and classification of diabetes mellitus and its complications. Part 1: diagnosis and classification of diabetes mellitus provisional report of a WHO consultation. Diabet Med. 1998;15:539‐553. 9686693 10.1002/(SICI)1096-9136(199807)15:7<539::AID-DIA668>3.0.CO;2-S

[9] Wilkinson CP , Ferris FR , Klein RE , et al. Proposed international clinical diabetic retinopathy and diabetic macular edema disease severity scales. Ophthalmology. 2003;110:1677‐1682. 13129861 10.1016/S0161-6420(03)00475-5

[10] Barr CC . Retinopathy and nephropathy in patients with type 1 diabetes four years after a trial of intensive insulin therapy. N Engl J Med. 2000;342:381‐389. 10666428 10.1056/NEJM200002103420603 PMC2630213

[11] White NH , Sun W , Cleary PA , et al. Effect of prior intensive therapy in type 1 diabetes on 10‐year progression of retinopathy in the DCCT/EDIC: comparison of adults and adolescents. Diabetes. 2010;59:1244‐1253. 20150283 10.2337/db09-1216 PMC2857905

[12] Aiello LP . Diabetic retinopathy and other ocular findings in the diabetes control and complications trial/epidemiology of diabetes interventions and complications study. Diabetes Care. 2014;37:17‐23. 24356593 10.2337/dc13-2251 PMC3867989

[13] Lachin JM , White NH , Hainsworth DP , Sun W , Cleary PA , Nathan DM . Effect of intensive diabetes therapy on the progression of diabetic retinopathy in patients with type 1 diabetes: 18 years of follow‐up in the DCCT/EDIC. Diabetes. 2015;64:631‐642. 25204977 10.2337/db14-0930 PMC4303965

[14] Fullerton B , Jeitler K , Seitz M , Horvath K , Berghold A , Siebenhofer A . Intensive glucose control versus conventional glucose control for type 1 diabetes mellitus. Cochrane Database Syst Rev. 2014;(2):CD009122. https://doi.org./10.1002/14651858.CD009122.pub2. 10.1002/14651858.CD009122.pub2 PMC6486147 24526393

[15] Swedish Council on Health Technology Assessment . Intensive Glucose‐Lowering Therapy in Diabetes: A Systematic Review. Stockholm, Sweden: Swedish Council on Health Technology Assessment; 2009. 28876798

[16] Masser DR , Vanguilder SH , Bixler GV , Dunton W , Bronson SK , Freeman WM . Insulin treatment normalizes retinal neuroinflammation but not markers of synapse loss in diabetic rats. Exp Eye Res. 2014;125:95‐106. 24931083 10.1016/j.exer.2014.06.005 PMC4122586

[17] Monnier L , Colette C , Owens DR . Glycemic variability: the third component of the dysglycemia in diabetes. Is it important? How to measure it? J Diabetes Sci Technol. 2008;2:1094‐1100. 19885298 10.1177/193229680800200618 PMC2769808

[18] The relationship of glycemic exposure (HbA1c) to the risk of development and progression of retinopathy in the diabetes control and complications trial. Diabetes. 1995;44:968‐983. 7622004

[19] Gimeno‐Orna JA , Castro‐Alonso FJ , Boned‐Juliani B , Lou‐Arnal LM . Fasting plasma glucose variability as a risk factor of retinopathy in type 2 diabetic patients. J Diabetes Complications. 2003;17:78‐81. 12614973 10.1016/s1056-8727(02)00197-6

[20] Mohsin F , Craig ME , Cusumano J , et al. Discordant trends in microvascular complications in adolescents with type 1 diabetes from 1990 to 2002. Diabetes Care. 2005;28:1974‐1980. 16043741 10.2337/diacare.28.8.1974

[21] Takao T , Ide T , Yanagisawa H , Kikuchi M , Kawazu S , Matsuyama Y . The effect of fasting plasma glucose variability on the risk of retinopathy in type 2 diabetic patients: retrospective long‐term follow‐up. Diabetes Res Clin Pract. 2010;89:296‐302. 20416966 10.1016/j.diabres.2010.03.027

[22] Hietala K , Waden J , Forsblom C , et al. HbA1c variability is associated with an increased risk of retinopathy requiring laser treatment in type 1 diabetes. Diabetologia. 2013;56:737‐745. 23314044 10.1007/s00125-012-2816-6

[23] Hermann JM , Hammes HP , Rami‐Merhar B , et al. on behalf of the DPV Initiative the German BMBF Competence Network Diabetes Mellitus. HbA1c variability as an independent risk factor for diabetic retinopathy in type 1 diabetes: a German/Austrian multicenter analysis on 35 891 patients. PLoS One. 2014;9:e91137. 24609115 10.1371/journal.pone.0091137 PMC3946653

[24] Virk SA , Donaghue KC , Cho YH , et al. Association between HbA1c variability and risk of microvascular complications in adolescents with type 1 diabetes. J Clin Endocrinol Metab. 2016;101:3257‐3263. 27186858 10.1210/jc.2015-3604

[25] Joussen AM , Poulaki V , Le ML , et al. A central role for inflammation in the pathogenesis of diabetic retinopathy. FASEB J. 2004;18:1450‐1452. 15231732 10.1096/fj.03-1476fje

[26] Rubsam A , Parikh S , Fort PE . Role of inflammation in diabetic retinopathy. Int J Mol Sci. 2018;19 pii: E942. https://doi.org./10.3390/ijms19040942. 10.3390/ijms19040942 PMC5979417 29565290

[27] Quagliaro L , Piconi L , Assaloni R , Martinelli L , Motz E , Ceriello A . Intermittent high glucose enhances apoptosis related to oxidative stress in human umbilical vein endothelial cells: the role of protein kinase C and NAD(P)H‐oxidase activation. Diabetes. 2003;52:2795‐2804. 14578299 10.2337/diabetes.52.11.2795

[28] Piconi L , Quagliaro L , Da RR , et al. Intermittent high glucose enhances ICAM‐1, VCAM‐1, E‐selectin and interleukin‐6 expression in human umbilical endothelial cells in culture: the role of poly(ADP‐ribose) polymerase. J Thromb Haemost. 2004;2:1453‐1459. 15304054 10.1111/j.1538-7836.2004.00835.x

[29] Quagliaro L , Piconi L , Assaloni R , et al. Intermittent high glucose enhances ICAM‐1, VCAM‐1 and E‐selectin expression in human umbilical vein endothelial cells in culture: the distinct role of protein kinase C and mitochondrial superoxide production. Atherosclerosis. 2005;183:259‐267. 16285992 10.1016/j.atherosclerosis.2005.03.015

[30] Sun J , Xu Y , Sun S , Sun Y , Wang X . Intermittent high glucose enhances cell proliferation and VEGF expression in retinal endothelial cells: the role of mitochondrial reactive oxygen species. Mol Cell Biochem. 2010;343:27‐35. 20524146 10.1007/s11010-010-0495-5

[31] Zhou J , Wang S , Xia X . Role of intravitreal inflammatory cytokines and angiogenic factors in proliferative diabetic retinopathy. Curr Eye Res. 2012;37:416‐420. 22409294 10.3109/02713683.2012.661114

[32] Hussein KA , Choksi K , Akeel S , et al. Bone morphogenetic protein 2: a potential new player in the pathogenesis of diabetic retinopathy. Exp Eye Res. 2014;125:79‐88. 24910902 10.1016/j.exer.2014.05.012 PMC4122600

[33] Ohno‐Matsui K , Yoshida T , Uetama T , Mochizuki M , Morita I . Vascular endothelial growth factor upregulates pigment epithelium‐derived factor expression via VEGFR‐1 in human retinal pigment epithelial cells. Biochem Biophys Res Commun. 2003;303:962‐967. 12670505 10.1016/s0006-291x(03)00446-7

[34] Zhang SX , Wang JJ , Gao G , Parke K , Ma JX . Pigment epithelium‐derived factor downregulates vascular endothelial growth factor (VEGF) expression and inhibits VEGF‐VEGF receptor 2 binding in diabetic retinopathy. J Mol Endocrinol. 2006;37:1‐12. 16901919 10.1677/jme.1.02008

[35] Qiu AW , Bian Z , Mao PA , Liu QH . IL‐17A exacerbates diabetic retinopathy by impairing Muller cell function via Act1 signaling. Exp Mol Med. 2016;48:e280. 27980343 10.1038/emm.2016.117 PMC5192073

[36] Karbasforooshan H , Karimi G . The role of SIRT1 in diabetic retinopathy. Biomed Pharmacother. 2018;97:190‐194. 29091865 10.1016/j.biopha.2017.10.075

[37] Afzal N , Zaman S , Asghar A , et al. Negative association of serum IL‐6 and IL‐17 with type‐II diabetes retinopathy. Iran J Immunol. 2014;11:40‐48. 24632587 10.22034/iji.2014.16764

[38] Nadeem A , Javaid K , Sami W , et al. Inverse relationship of serum IL‐17 with type‐II diabetes retinopathy. Clin Lab. 2013;59:1311‐1317. 24409666 10.7754/clin.lab.2013.121140

[39] Dandona P , Chaudhuri A , Ghanim H , Mohanty P . Insulin as an anti‐inflammatory and antiatherogenic modulator. J Am Coll Cardiol. 2009;53(suppl):S14‐S20. 19179212 10.1016/j.jacc.2008.10.038

